# Burnout, resilience and the quality of life among Malaysian healthcare workers during the COVID-19 pandemic

**DOI:** 10.3389/fpubh.2022.1021497

**Published:** 2022-11-30

**Authors:** Roy Rillera Marzo, Yassmein Khaled, Mohamed ElSherif, Muhd Siv Azhar Merican Bin Abdullah, Hui Zhu Thew, Collins Chong, Shean Yih Soh, Ching Sin Siau, Shekhar Chauhan, Yulan Lin

**Affiliations:** ^1^International Medical School, Management and Science University, Shah Alam, Selangor, Malaysia; ^2^Global Public Health, Jeffrey Cheah School of Medicine and Health Sciences, Monash University Malaysia, Subang Jaya, Selangor, Malaysia; ^3^Department of Epidemiology and Population Health, Faculty of Health Sciences, American University of Beirut, Beirut, Lebanon; ^4^Department of Community Health, Faculty of Medicine, Universiti Kebangsaan Malaysia, Bangi, Selangor, Malaysia; ^5^Department of Family Medicine, Faculty of Medicine and Health Sciences, Universiti Putra Malaysia, Serdang, Selangor, Malaysia; ^6^Department of Anaesthesia and Intensive Care, Faculty of Medicine and Health Sciences, Universiti Putra Malaysia, Serdang, Selangor, Malaysia; ^7^Department of Psychiatry, Faculty of Medicine and Health Sciences, Universiti Putra Malaysia, Serdang, Selangor, Malaysia; ^8^Centre for Community Health Studies, Faculty of Health Sciences, Universiti Kebangsaan Malaysia, Kuala Lumpur, Malaysia; ^9^Department of Family and Generations, International Institute for Population Sciences, Mumbai, India; ^10^Department of Epidemiology and Health Statistics, School of Public Health, Fujian Medical University, Fuzhou, China

**Keywords:** burnout, resilience, quality of life, health-workers, COVID-19

## Abstract

**Background:**

Healthcare workers have to deal with highly demanding work situations, making healthcare as one of the most challenging professions. Up to now, far too little attention has been paid to burnout, resilience and the quality of life among Malaysian healthcare workers. Therefore, this paper explores the correlation between burnout, resilience and quality of life among Malaysian healthcare workers during the COVID-19 pandemic.

**Method:**

A total of 394 healthcare workers reported their responses on Maslach Burnout Inventory questionnaire, World Health Organization Quality of Life (WHOQOL)-BREF, and Brief Resilience Scale. Respondents were contacted through convenience sampling method and targeted population constituted Malaysian healthcare workers aged 18 years and above.

**Results:**

For occupational exhaustion, about 50.5% of participants have moderate degree, 40.6% have high degree, and 8.9% have low degree of burnout. Health workers from age 25 to 35 years have lower physical health compared to health workers aged <25 years (coefficient = −0.77, *p* = 0.021). Similarly, healthcare workers who were working more than 10 h every day were more likely to report poor psychological health (coefficient = −2.49, *p* = 0.06). Positive correlation between physical and psychological health was observed. Further, a negative correlation was found between occupational exhaustion and the quality of life.

**Conclusion:**

It is important to target physical as well as psychological wellbeing of the healthcare workers. Also, it is important to understand the contribution of long working hours in declining the quality of life of the healthcare workers. Thus, allocating fixed working hours for healthcare workers would bring a much-required change.

## Introduction

Healthcare workers have to deal with highly demanding work situations, making healthcare as one of the most challenging professions. As a result of the highly stressful nature of the healthcare profession, it requires the healthcare workers to possess a high level of physical and emotional resilience to deal with work-related issues to perform better at work (1). Therefore, the quality of one's mental health determines the capacity of an individual to perform their roles, including their ability to work. The state of good mental health is defined by feeling well and coping well with pressure, adapting to changing conditions, enjoying rewarding relationships and performing duties effectively (2).

It is well-known that stress is a physiological response to pressure in a wide range of situations and events in our lives (3). It is often triggered when we are faced with something unexpected or new in our lives or feel that we have little control over the situation. Our responses to stress differ from one another. Depending on our genetics, our early life experiences, our personalities, and our economic and social circumstances, we all cope with different situations to varying degrees. Our body reacts to stress by producing stress hormones that are responsible for triggering a fight or flight response when faced with stress. This helps us respond quickly to dangerous situations. Getting through fear or pain is sometimes essential for us to succeed in an endeavor and is unlikely that our stress hormones will remain elevated for long after a stressful event has passed. There can, however, be negative effects if too much stress is experienced. Eventually we can end up in a permanent state of fight or flight, overburdened or unable to cope. In the long run, this can have a negative effect on our physical and mental health and lead to disorder.

According to the DSM-5, acute stress disorder is characterized by specific fear behaviors that last from 3 days up to 1 month following a traumatic event (4). The symptoms are always triggered by death, serious injury, or sexual assault. The DSM-5 lists physical attack, physical abuse, mugging, active combat, sexual violence, natural disasters, and serious accidents as traumatic events. Traumatic events or hearing about the violent death of a loved one can also trigger acute stress disorder. It affects every aspect of a patient's life. Depressive symptoms are associated with acute stress disorder, and causes problems with feelings of joy, happiness, satisfaction, and sexual arousal in patients. The negative impact also may lead to being late for work and appointments or missing them altogether. In turn, this will cause sleep problems, which can further lead to mood issues as well as a lack of energy and focus. As these conditions persist on a long-term basis, there are dangers that impulsive behavior, such as gambling, substance abuse, or reckless driving, could occur because of these behaviors. It is extremely common for people to turn to drugs and alcohol after experiencing trauma. It is possible that alcohol or drug abuse may persist after the designated time for acute stress disorder has expired in some cases (5).

However, long-term exposure to work-related stress can cause burnout (2). The term burnout was first introduced in 1974 by Freudenberger due to a professional's physical and mental exhaustion (6). The World Health Organization (WHO) has recently updated the definition of burnout as a chronic stress condition associated with prolonged periods of high-stress levels and is, therefore, an occupational phenomenon (7). WHO defines quality of life as the perception of an individual's life position in relation to the culture, emphasizing that quality of life is subjective that includes both positive and negative facets of life and is multidimensional in nature.

According to the International Classification of Diseases, 11th edition, burnout is an occupational phenomenon associated with employment or unemployment that causes the person to feel tired or drained of energy. Cynicism or negativism about one's job, or an increase in mental distance from it. A reduction in professional effectiveness. The syndrome is specific to work environments and should not be applied elsewhere (8).

Worldwide, there has been an increase in burnout following the outbreak of coronavirus disease 2019 (COVID-19) that resulted in increasing the number of competing demands on the healthcare systems (9–11). In Malaysia, healthcare workers are reported to be more likely to suffer from burnout due to long working hours, lack of social support from their families, and stressful work conditions that leads to high rates of job dissatisfaction and worker migration to other countries (12). Since the spread of COVID-19, burnout has escalated and worsened in many areas of healthcare. Consequently, it negatively impacts the quality of life in a wide range of individuals, as well as quality of care for patients leading to higher levels of emotional exhaustion and turnover for those delivering care to patients (10).

Resilience was reported to be a significant predictor of compassion satisfaction, secondary traumatic stress and burnout as possible protective factors against burnout (13, 14). Recent studies have also described resilience as a critical quality that allows healthcare workers to face challenges amid the COVID-19 pandemic (15). The concept of resilience refers to a dynamic process of positive adaptation in the face of stress or adversity. Experience, learning and training can all contribute to developing this skill. The ability to recover or bounce back from adverse circumstances distinguishes resilient individuals (13, 16, 17).

Healthcare workers or healthcare professionals are provider of healthcare treatment and these can include nurse, physician, internist, surgeon, radiologist, obstetrician, psychiatrist optometrist, medical assistant, midwife, dentist and so on based on their qualification and expertise. Each one of the healthcare professional has different role with varying work burden and therefore it is possible that one type of healthcare professional may feel burnout while other may not and similarly, one may have higher resilience than other healthcare professionals because of different training they have undergone to become one of the healthcare professionals. Doctors and nurses were put under extreme work burden during COVID-19 pandemic and that is why they had poor emotional experience including depression, burnout, and anxiety (18). Tabur et al. (18) in their study noted that around two-fifths (38.6%) of the nurses/midwives and one-sixth (17.3%) of the doctors experienced severe burnout (18). Adequate preparedness for response to COVID-19 was found to be associated with lower burnout among healthcare workers (19). Further, appreciation from management and family support helped healthcare workers in managing their burnout and a study found that these factors were associated with lower burnout risk among healthcare professionals (19). Resilience and burnout go hand in hand as these are interdependent, Di Trani et al. (20), in their study conducted among Italian healthcare workers, noted that high-risk burnout group had lower resilience and greater difficulties in tolerating the uncertainties than the low-risk burnout group (20).

Since the coronavirus pandemic erupted in late 2019, several studies have explored the knowledge, attitude and practices and ill-effects of COVID-19 on population health (21–28). After the advent of COVID-19 vaccines, studies also examined the vaccine hesitancy and effectiveness among various population (29–33). Also, quality of life during COVID-19 pandemic has been reported in abundance (34, 35). In addition, some studies have also explored mental health, risk perception, and coping strategies adopted by healthc are workers during COVID-19 pandemic (36–38). Moreover, Psychological support is largely underutilized due to a lack of awareness, equipment, staff time, or skill for intervention, according to systematic reviews. On the other hand, effective communication, safe and supportive environments for frontline workers, and careful attention to local needs can promote mental health (39).

However, studies examining burnout, resilience, and quality of life altogether are missing in the Malaysian context. Research on early burnout has focused on maintaining the integrity of healthcare workers' mental health. This has emerged as a crucial topic throughout the COVID-19 pandemic and has been receiving attention as a key to preventing the progression of mental disorders (40, 41). A high level of burnout has a negative impact on the healthcare industry, healthcare services, as well as healthcare workers. Little is known about burnout, resilience and the quality of life among healthcare workers in Malaysia, and it is unclear what factors are associated with it (42). This paper explores the correlation between burnout, resilience and quality of life among Malaysian healthcare workers during the COVID-19 pandemic.

## Materials and methods

### Study population and sampling

This cross-sectional study was conducted between February 15, 2022 and March 15, 2022 to evaluate the level of burnout, quality of life and resilience among healthcare workers from Putrajaya and Selangor hospitals. The study was conducted during the fifth wave fuelled by the Omicron variant that led to maximum daily cases in February and March 2022 (43, 44), but is marked by lower numbers of hospitalizations and deaths than during the spread of the Delta variant (44). In March 2022, the BA.2 Omicron sub-variant is projected to be the dominant strain in the country (45). The country's vaccination programme, which commenced in late February 2021 (46), has fully inoculated over 80% of the population and 97% of adults as of 24 April 2022 (47). On February 13, 2022, the total number of cases in Malaysia exceeded the 3 million mark, reaching 3,040,235 (47). By February 24, 2022, the total number of recoveries had reached the 3 million mark, reaching 3,018,172 (47).

The study used a convenience sampling method for recruitment. The online survey was disseminated *via* various social media platforms such as Instagram, Twitter, LinkedIn, Facebook, WhatsApp, and Telegram. The target population was adult Malaysian healthcare workers aged 18 years and above. We invited Malaysian assistant medical officers, doctors, health inspectors, hospital food preparation personnel, medical laboratory technologists, nurses, paramedics, pharmacists, physicians, physiotherapists, dieticians, therapists, psychologists, counselors, radiographers, and social workers from public and private healthcare services to enroll in this study. All respondents were informed that their participation was anonymous and voluntary at the beginning of the survey. Consent was implied if the participants started answering the questionnaire. This research complied with the tenets of the Declaration of Helsinki.

Cochran's formula was used to calculate the minimum recommended sampling size (48). The minimal sample size required for this study, with a confidence level of 95%, ± 5% precision and 0.5 estimated proportion, was 385 study participants. A total of 394 completed responses were collected.

The Institutional Review Board granted approval (Above already mention). Participation was voluntary and anonymity was assured. All personal information was kept confidential. Furthermore, researchers analyzed only de-identified data.

### Study instruments

#### Sociodemographic and work-related characteristics

The data collection instrument comprised of five parts. The first part of the tool asked questions pertaining sociodemographic and work-related characteristics. The choice of variables was informed by the available literature and inputs from the investigators. Participants were requested to indicate their age, gender, marital status, speciality, educational level, income, number of family members, job title, place of work, years of experience, hours of working, and socialization time per week. This section also asked whether the respondent had been attending COVID-19 patients directly, had been infected with COVID-19, their willingness to having COVID-19 vaccine's booster doses in the future.

#### Maslach burnout inventory questionnaire

The second part of the study tool was a translated version of Maslach Burnout Inventory (MBI) (49). To limit the study to burnout related to COVID-19, the phrase “due to COVID-19” was added to each item. MBI is an internationally recognized, validated, self-report questionnaire for measuring the severity of workplace burnout, using the three dimensions of emotional exhaustion, depersonalization and personal accomplishment. The questionnaire has 22 items and each item is answered on a seven-point Likert scale. This tool has been extensively used in many studies in different parts of the world and the Malay translation has also been validated previously (50, 51).

Burnout is expressed by scores of each of the three MBI subscales, with a high score meaning a high level of burnout. Each subscale score is calculated by adding up all scores of all items in that subscale, with the notion that the items on personal accomplishment domain are reversely scored (49). Scores range from 0 to 54 for emotional exhaustion (EE), from 0 to 30 for depersonalization (DP) and from 0 to 48 for personal accomplishment (PA) subscale. Scales are scored such that higher scores indicate more of each construct. Higher scores on the EE and DP subscales indicate a higher burnout symptom burden; lower scores on the PA subscale indicate a higher burnout symptom burden. The standard cut-off values were used to define low, moderate, and high levels in each dimension (49).

#### WHO quality of life-BREF 26

The WHO quality of life (WHOQOL)-BREF is a 26-item instrument consisting of four domains: physical health (7 items), psychological health (6 items), social relationships (3 items) and environmental health (8 items); it also contains QOL and general health items. Each individual item of the WHOQOL-BREF is scored from 1 to 5 on a response scale, which is stipulated as a five-point ordinal scale.

The physical health domain questions are based on daily activities, medical aid, energy, mobility, the extent of pain, sleeping pattern and working capacity. The psychological domain focuses on participants' personal beliefs, positive and negative feelings, self-esteem, body image, thoughts and learning capabilities. The social relationships domain explores the respondent's overall satisfaction with their personal and social life. Lastly, the environmental domain comprises questions about safety and security, contentment with one's property and physical surroundings, finances (does one have enough money to satisfy one's requirements), access to the necessary care, information and transport. Moreover, the questionnaire has two specific questions regarding participants' opinions regarding their overall quality of life and health. We used the Malay validated version of the original WHOQOL-BREF questionnaire (52, 53).

#### Brief resilience scale

The last section is the Brief Resilience Scale (BRS) questionnaire to assess the perceived ability to bounce back or recover from stress. The scale was developed to assess a unitary construct of resilience, including both positively and negatively worded items.

The Brief Resilience Scale has six items presented in Table 1. Items 1, 3, and 5 are positively worded, and items 2, 4, and 6 are negatively worded. The BRS is scored by reverse coding items 2, 4, and 6 and finding the mean of the six items. The following instructions are used to administer the scale: “Please indicate the extent to which you agree with each of the following statements by using the following scale: 1 = strongly disagree, 2 = disagree, 3 = neutral, 4 = agree, 5 = strongly agree.” The possible score range on the BRS is from 1 (low resilience) to 5 (high resilience). It composes of 6 questions with a score on interpretation 1.00–2.99 as low resilience, 3.00–4.30 as normal resilience and 4.31–5.00 as high resilience.

**Table 1:** Socio-demographics of participants (*N* = 394).

	**N**	**%**
Gender		
Male	51	12.9
Female	343	87.1
Age		
< 25 years	81	20.6
From 25 to 35 years	170	43.1
From 35 to 55 years	123	31.2
More than 55years	20	5.1
Specialty		
Doctor	55	14.0
Nurse	248	62.9
Others	91	23.1
Income level		
**≤**Less than RM 4,850	282	71.6
RM 4,850–10,959	100	25.4
**≥** RM 10,959	12	3.0
Education background		
Primary	30	7.6
Secondary	178	45.2
Tertiary	186	47.2
Working experiences		
No experience	28	7.1
<5 years	125	31.7
From 5 to 10 years	92	23.4
More than 10 years	149	37.8
Working duration		
<7 h	4	1.0
7 h daily	117	29.7
From 8 to 10 h daily	216	54.8
More than 10 h	57	14.5
Family members		
<5	152	38.6
5–10	220	55.8
More than 10	22	5.6
Socializing duration		
No time	12	3.0
<5 h	95	24.1
From 5 to 10 h	129	32.7
More than 10 h	158	40.1
Dealing with COVID-19		
No	205	52.0
Yes	189	48.0
Infected with COVID-19		
No	213	54.1
Yes	181	45.9
COVID-19 vaccine booster		
Very unlikely	32	8.1
Unlikely	63	16.0
Somewhat unlikely	58	14.7
Somewhat likely	79	20.1
Likely	101	25.6
Very likely	61	15.5

### Data analysis

Descriptive statistics are presented in the form of frequencies and percentages for the categorical variables. Mean and standard deviation (SD) are reported for numerical variables. Multiple linear regression was used to study the association of different variables with the outcomes of interest. Correlation between quality of life, burnout, and resilience levels was studied using Pearson's correlation and a heatmap for the correlation coefficients was developed. IBM SPSS 28 for windows software was used for the analysis while Stata 17 software was used for the multiple linear regression. *P* < 0.05 is considered statistically significant.

### Ethic statement

The study was designed and conducted in line with the declaration of Helsinki and was approved by the Ethics Committee of Management and Science University (Ethics Code: MSU-RMC-02/FR01/09/L1/085). Respondents were informed that their participation was voluntary, and written consent was implied on the completion of the questionnaire. All participants were aged 18 years or older.

## Results

A total of 394 participants were enrolled in this study. About 87.1% of them were females, while the rest were males (Table 1). 43.1% of the participants belonged to the age group of 25–35 years old, 31.2% were 36 to 55 years old, 20.6% were <25 years, and 5.1% were more than 55 years old. About 62.9% of participants were nurses, 14% were doctors and the rest were from other specialties in the medical field. About 71.6% of participants' income was less than RM 4,850, 25.4% of participants' income was from RM 4,850 to RM 10,959, while the rest took more than RM 10,959 per month. About 47.2% of participants had tertiary education, 45.2% of them had secondary education, while the rest had primary education. About 37.8% of the participants had work experience of more than 10 years, 31.7% had work experience of <5 years, 23.4% had work experience from 5 to 10 years and 7.1% did not have any experience. About 54.8% of participants worked from 8 to 10 hours daily, 29.7% worked 7 h daily, 14.5% worked more than 10 h daily and 1% worked <7 h daily. About 40.1% of participants were socializing more than 10 h weekly, 32.7% were socializing from 5 to 10 h and 24.1% were socializing <5 h weekly, while the rest did not socialize at all. Of all the participants, 45.9% had been infected with COVID-19.

For occupational exhaustion, about 50.5, 40.6, and 8.9% of the participants had moderate, high and low degree of burnout, respectively (Table 2). For degree of depersonalization, high, moderate and low degree of burnout was found in 89.1, 9.6, and 1.3% of participants, respectively. For D]degree of personal accomplishment, about 76.4% of participants have low degree, 15% have moderate degree, and 8.6% have high degree of burnout.

**Table 2:** Maslach burnout inventory questionnaire.

	**Mean**	**SD**
Occupational exhaustion	27.84	7.05
I feel emotionally exhausted because of my work.	3.55	1.60
I feel worn out at the end of a working day.	3.52	1.63
I feel tired as soon as I get up in the morning and see a new working day stretched out in front of me.	3.36	1.67
Working with people the whole day is stressful for me.	2.55	1.74
I feel burned out because of my work.	3.23	1.75
I feel frustrated by my work.	2.47	1.72
I get the feeling that I work too hard.	3.49	1.65
Being in direct contact with people at work is too stressful.	2.58	1.74
I feel as if I'm at my wits end.	2.65	1.66
Degree of depersonalization	17.10	4.72
I get the feeling that I treat some clients/colleagues impersonally, as if they were objects.	2.42	1.78
I have become more callous to people since I have started doing this job.	2.62	1.73
I'm afraid that my work makes me emotionally harder.	2.93	1.74
I'm not really interested in what is going on with many of my colleagues.	2.59	1.79
I have the feeling that my colleagues blame me for some of their problems.	2.31	1.75
Degree of personal accomplishment	26.49	9.55
I can easily understand the actions of my colleagues/supervisors.	4.07	1.48
I deal with other people's problems successfully.	3.60	1.49
I feel that I influence other people positively through my work.	3.69	1.49
I feel full of energy.	3.58	1.55
I find it easy to build a relaxed atmosphere in my working environment.	3.32	1.52
I feel stimulated when I had been working closely with my colleagues.	3.57	1.57
I have achieved many rewarding objectives in my work.	3.26	1.62
In my work, I am very relaxed when dealing with emotional problems.	3.23	1.47

The means and SD for each question and domain are reported in Table 3.

**Table 3:** (WHOQOL-BREF) questionnaire.

	**Mean**	**SD**
**Physical health**	12.78	2.33
To what extent do you feel that physical pain prevents you from doing what you need to do? (in the last 2 weeks)	2.92	1.06
How much do you need any medical treatment to function in your daily life? (in the last 2 weeks)	2.53	1.17
Do you have enough energy for everyday life?	3.36	0.97
How well are you able to get around physically?	3.40	0.90
How satisfied are you with your sleep?	3.19	1.11
How satisfied are you with your ability to perform your daily living activities?	3.49	0.91
How satisfied are you with your capacity for work?	3.47	0.94
**Psychological health**	13.5	2.5
How much do you enjoy life? (in the last 2 weeks)	3.40	1.03
To what extent do you feel your life to be meaningful? (in the last 2 weeks)	3.54	0.95
How well are you able to concentrate?	3.46	0.86
Are you able to accept your bodily appearance?	3.41	1.01
How satisfied are you with yourself?	3.55	1.05
How often do you have negative feelings such as blue mood, despair, anxiety or depression?	2.91	1.15
**Environment**	14.1	3.35
How safe do you feel in your daily life?	3.56	0.86
How healthy is your physical environment?	3.40	0.96
Do you have enough money to meet your needs?	3.29	1.11
How available to you is the information you need in your daily life?	3.57	0.84
To what extent do you have the opportunity for leisure activities?	3.19	0.92
How satisfied are you with the conditions of your living place?	3.76	0.91
How satisfied are you with your access to health services?	3.77	0.91
How satisfied are you with your transport?	3.91	0.94
**Social relationships**	14.2	2.6
How satisfied are you with your personal relationships?	3.65	1.01
How satisfied are you with your sex life?	3.35	1.10
How satisfied are you with the support you get from your friends?	3.64	0.94

Table 4 depicts the mean and SD for BRS questionnaire, about 23.9% of participants have low resilience, 74.6% have normal resilience, and 1.5% has high resilience.

**Table 4:** The brief resilience scale questionnaire.

	**Mean**	**SD**
I tend to bounce back quickly after hard times	3.46	0.86
I have a hard time making it through stressful events.	2.88	0.94
It does not take me long to recover from a stressful event.	3.28	0.97
It is hard for me to snap back when something bad happens.	2.92	0.97
I usually come through difficult times with little trouble.	3.07	0.94
I tend to take a long time to get over setbacks in my life.	2.97	0.97

Multiple linear regressions were done to study the factors associated with physical health (Table 5). The only significant factor was age. Healthcare workers from age 25 to 35 years have lower physical health compared to healthcare workers < 25 years (coefficient = −0.77, *P* = 0.021).

**Table 5:** Multiple linear regression for the factors associated with physical health.

	**Coefficient**	**P-value**	**95% C.I. of the coefficient**	
Age				
< 25 years				
From 25 to 35 years	−0.77	0.021	−1.43	−0.12
From 35 to 55 years	−0.17	0.646	−0.90	0.56
More than 55 years	−0.37	0.561	−1.64	0.89
Gender				
Male				
Female	0.07	0.850	−0.63	0.77
Specialty				
Doctor				
Nurse	0.60	0.181	−0.28	1.48
Others	0.10	0.825	−0.78	0.98
Education level				
Primary				
Secondary	0.30	0.524	−0.62	1.21
Tertiary	0.03	0.956	−0.94	1.00
Income level				
**≤** RM 4,850				
RM 4,850–10,959	0.07	0.83	−0.58	0.72
**≥** RM 10,959	0.53	0.52	−1.08	2.13
Working duration				
< 7 h				
7 h daily	−0.52	0.663	−2.86	1.82
From 8 to 10 h daily	−0.45	0.707	−2.77	1.88
More than 10 h	−1.70	0.163	−4.08	0.69
Socializing duration				
No time				
< 5 h	0.03	0.964	−1.41	1.48
From 5 to 10 h	0.14	0.845	−1.29	1.57
More than 10 h	0.28	0.700	−1.14	1.70

Multiple linear regressions were done to study the factors associated with psychological health (Table 6). The only significant factors were educational level and working duration. Health workers who have secondary education level had higher psychological health compared to health workers have primary education level (coefficient = 1.0, *P* = 0.049). Similarly, participants who were working more than 10 h every day were more likely to report poor psychological health.

**Table 6:** Multiple linear regression for the factors associated with psychological health.

	**Coefficient**	**P-value**	**95% C.I. of the coefficient**	
Age				
< 25				
From 25 to 35 years	−0.49	0.178	−1.20	0.22
From 35 to 55	0.00	0.994	−0.80	0.79
More than 55	0.03	0.97	−1.35	1.41
Gender				
Male				
Female	0.27	0.491	−0.49	1.03
Specialty				
Doctor				
Nurse	0.61	0.214	−0.35	1.56
Others	0.38	0.43	−0.57	1.34
Education level				
Primary				
Secondary	1.00	0.049	0.00	1.99
Tertiary	0.54	0.315	−0.52	1.60
Income level				
**≤**RM 4,850				
RM 4850–10,959	0.28	0.432	−0.42	0.99
**≥** RM 10,959	1.29	0.148	−0.46	3.04
Working duration				
< 7 h				
7 h daily	−1.60	0.218	−4.15	0.95
From 8 to 10 h daily	−1.51	0.241	−4.05	1.02
More than 10 h	−2.49	0.06	−5.09	0.11
Socializing duration				
No time				
< 5 h	0.50	0.529	−1.07	2.08
From 5 to 10 h	0.69	0.384	−0.87	2.24
More than 10 h	0.74	0.345	−0.80	2.29

Table 7 depicts result for multiple linear regressions for social relationships. None of the background variables were significant predictor for social relationships.

**Table 7:** Multiple linear regressions for the factors associated with social relationships.

	**Coefficient**	**P-value**	**95% C.I. of the coefficient**	
Age				
< 25 years				
From 25 to 35 years	0.48	0.328	−0.48	1.43
From 35 to 55 years	0.60	0.27	−0.47	1.67
More than 55 years	0.66	0.481	−1.19	2.52
Gender				
Male				
Female	−0.32	0.538	−1.34	0.70
Specialty				
Doctor				
Nurse	1.07	0.103	−0.22	2.35
Others	0.37	0.572	−0.91	1.65
Education level				
Primary				
Secondary	0.86	0.205	−0.47	2.19
Tertiary	0.14	0.847	−1.28	1.56
Income level				
**≤** RM 4,850				
RM 4850–10,959	0.44	0.356	−0.50	1.39
**≥** RM 10,959	1.54	0.198	−0.81	3.88
Working duration				
< 7 h				
7 h daily	−0.46	0.791	−3.88	2.96
From 8 to 10 h daily	−0.47	0.784	−3.88	2.93
More than 10 h	−1.65	0.353	−5.14	1.84
Socializing duration				
No time				
< 5 h	−0.32	0.763	−2.43	1.79
From 5 to 10 h	0.22	0.834	−1.86	2.31
More than 10 h	0.47	0.652	−1.60	2.55

Multiple linear regressions were done to study the factors associated with environment level (Table 8). The only significant factor was income level. Health workers who had Rm 4,850–10,959 have higher environmental level compared to health workers who had < Rm 4,850 (coefficient = 0.81), *P* = 0.028. Health workers who have > Rm 10,959 have higher environment level compared to health workers have < Rm 4,850 (coefficient =1.92), *P* = 0.034.

**Table 8:** Multiple linear regressions for the factors associated with environment level.

	**Coefficient**	**P-value**	**95% C.I. of the coefficient**	
Age				
< 25 years				
From 25 to 35 years	−0.21	0.569	−0.94	0.52
From 35 to 55 years	0.42	0.308	−0.39	1.23
More than 55 years	0.88	0.219	−0.52	2.28
Gender				
Male				
Female	0.13	0.745	−0.65	0.90
Specialty				
Doctor				
Nurse	0.32	0.518	−0.65	1.29
Others	−0.11	0.824	−1.08	0.86
Education level				
Primary				
Secondary	0.63	0.224	−0.38	1.63
Tertiary	0.33	0.552	−0.75	1.40
Income level				
< Rm 4,850				
Rm 4,850–10,959	0.81	0.028	0.09	1.52
> Rm 10,959	1.92	0.034	0.15	3.70
Working duration				
< 7 h				
7 h daily	−1.81	0.172	−4.40	0.79
From 8 to 10 h daily	−1.60	0.222	−4.18	0.97
More than 10 h	−3.02	0.025	−5.66	−0.38
Socializing duration				
No time				
< 5 h	−0.47	0.56	−2.07	1.12
From 5 to 10 h	−0.12	0.882	−1.70	1.46
More than 10 h	0.14	0.864	−1.43	1.71

Multiple linear regressions were done to study the factors associated with level of resilience (Table 9). The only significant factor was gender. Participants aged more than 55 years have higher resilience compared to those aged <25 (coefficient = 0.391, *P* < 0.001). Participants who took more than Rm 10,959 have higher resilience compared to those taking <Rm 4,850 (coefficient = 0.458, *P* = 0.001).

**Table 9:** Multiple linear regression for the factors associated with level of resilience.

	**Coefficient**	**P-value**	**95% C.I. of the coefficient**	
Age				
< 25 years				
From 25 to 35 years	0.020	0.723	−0.091	0.131
From 35 to 55 years	−0.018	0.777	−0.142	0.106
More than 55 years	0.391	< 0.001	0.176	0.606
Gender				
Male				
Female	−0.050	0.407	−0.169	0.069
Specialty				
Doctor				
Nurse	0.130	0.086	−0.019	0.279
Others	0.114	0.134	−0.035	0.263
Education level				
Primary				
Secondary	−0.013	0.871	−0.168	0.142
Tertiary	−0.007	0.937	−0.172	0.158
Income level				
< Rm 4,850				
Rm 4,850–10,959	0.056	0.321	−0.054	0.165
> Rm 10,959	0.458	0.001	0.185	0.730
Working duration				
< 7 h				
7 h daily	0.021	0.916	−0.376	0.419
From 8 to 10 h daily	0.071	0.724	−0.324	0.466
More than 10 h	−0.018	0.930	−0.423	0.387
Socializing duration				
No time				
< 5 h	−0.025	0.839	−0.271	0.220
From 5 to 10 h	−0.050	0.686	−0.292	0.192
More than 10 h	0.040	0.744	−0.201	0.281

Table 10 depicts the correlation between quality of life, burnout and resilience levels. A negative correlation was found between occupational exhaustion and the four domains of quality of life; higher score in occupational exhaustion is associated with lower score in quality of life. Positive correlation was found between occupational exhaustion and resilience; higher score in occupational exhaustion is associated with high resilience level. Positive correlation was found between degree of depersonalization and resilience; higher score in degree of depersonalization was associated with high resilience level. A negative correlation was found between personal accomplishment degree and the four domains of quality of life; higher score in personal accomplishment degree is associated with lower score in quality of life. Negative correlation was found between personal accomplishment degree and resilience; higher score in personal accomplishment degree is associated with high resilience level. A positive correlation was found between resilience level and the four domains of quality of life; higher score in resilience level is associated with higher score in quality of life.

**Table 10:** Correlation between quality of life, burnout, and resilience levels.

	**Physical health**	**Psychological health**	**Social relationships**	**Environment level**	**EE score**	**DP**	**Pa**
Physical health	Pearson correlation	–						
Psychological health	Pearson correlation	0.812	–					
	p-value	< 0.001						
Social relationships	Pearson correlation	0.581	0.605	–				
	p-value	< 0.001	< 0.001					
Environment	Pearson correlation	0.751	0.739	0.651	–			
	p-value	< 0.001	< 0.001	< 0.001					
EE score	Pearson correlation	−0.136	−0.099	−0.224	−0.188	–		
	p-value	0.007	0.050	< 0.001	< 0.001				
DP	Pearson correlation	0.005	0.066	−0.045	0.030	0.615	–	
	p-value	0.914	0.194	0.375	0.549	< 0.001		
Pa	Pearson correlation	−0.252	−0.243	−0.277	−0.321	0.777	0.547	–
	p-value	< 0.001	< 0.001	< 0.001	< 0.001	< 0.001	< 0.001		
Resilience level	Pearson correlation	0.217	0.227	0.253	0.327	−0.150	0.052	−0.291
	p-value	< 0.001	< 0.001	< 0.001	< 0.001	0.003	0.299	< 0.001

Table 11 shows a heat map for the Correlation between quality of life, burnout, and resilience levels. The darker the color, the stronger is the correlation.

**Table 11:** Heatmap for correlation between quality of life, burnout, and resilience levels.

**Physical health**	**Psychological health**	**Social relationships**	**Environment**	**EE**	**DP**	**Pa**
Physical health	–						
Psychological health	0.746	–					
Social relationships	0.562	0.605	–				
Environment	0.722	0.739	0.651	–			
EE score	−0.112	−0.099	−0.224	−0.188	–		
DP	0.016	0.065	−0.044	0.030	0.615	–	
Pa	−0.216	−0.243	−0.277	−0.321	0.777	0.547	–
Resilience level	0.22	0.23	0.25	0.33	−0.15	0.05	−0.29
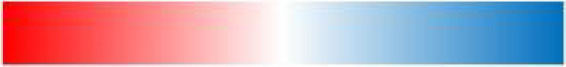							
−1	0	+1					

## Discussion

This study aimed to determine the factors that correlated with burnout, resilience, and quality of life among Malaysian healthcare workers during the COVID-19 pandemic. Approximately half of the healthcare workers in our study had a moderate to a high degree of burnout in occupational exhaustion and depersonalization with a low degree of personal accomplishment. According to the findings regarding quality of life, all four domains (physical health, psychological, social, and environmental) fell below the international standard (54). Of all the analyzed factors, age, education and income level correlated with physical health, psychological and environmental domains. The results of the study did not show any significant differences in the social domain. According to our study, all healthcare workers have an average level of resilience, and females demonstrated a lower level of resilience as compared to males. Systemic reviews have found that COVID-19 patients, psychiatric patients, health care workers, and the public all have mental health consequences during COVID-19 pandemic (55).

Many people across the globe are experiencing unexpected stress and trouble due to COVID-19. As a result, burnout is common among healthcare personnel who work in the COVID-19-affected healthcare system. Many variables, such as heavy workload, limited manpower and facilities to treat the rising number of patients and fear of contracting COVID-19 might be sources of burnout for the healthcare workers (56, 57). According to our study findings, over half of the Malaysian healthcare workers were burned out. These findings were similar to previous studies on healthcare workers in the neighboring countries (58, 59). Compared to the burnout rate before the pandemic, higher rates of burnout reported here indicate that the COVID-19 pandemic may have contributed to the increased staff burnout. During the pandemic crisis, we identified several characteristics positively connected to healthcare worker burnout. These characteristics included female gender, workplace, wage, workload, and finally, the COVID-19 status of a healthcare worker.

In our study, women appeared to be less resilient than men. They usually exhibit more significant emotional tiredness, posttraumatic stress, anxiety and depression than men, based on demographic and work-related characteristics. This result is comparable to the European countries (60, 61). Traditionally, females have been linked to a higher prevalence of these symptoms. This might be attributable to the fact that women have to spend more time in the family to take care of the family members instead of working outside. Most research conducted during the COVID-19 pandemic shows nurses worldwide have moderate resilience levels (62).

According to our study, our nurses have an average resilience score. Therefore, it is essential for healthcare professionals to prioritize assistance and other resources. Support and other resources must be available to people in front-line positions, especially during a pandemic, which presents unique challenges. Intervention should focus on increasing resilience in the health care personnel. These include communicating, evaluating burnout and offering assistance to those at risk for or experiencing burnout. Those healthcare workers who had gone beyond their resilience barrier should be provided with support and therapy before they safely return to work. Nobody should be stigmatized for seeking help for mental health. Mentors and peer support can effectively build a sense of coherence among healthcare workers. They are allowed to express their feelings and concerns openly (63). Constant expression of gratitude is a valuable strategy to promote resilience among health care workers (64).

One of the objectives of this study is to assess the quality of life led by healthcare workers during the COVID-19 pandemic crisis. This finding was consistent with a previous study, which showed that healthcare workers are below the international standard quality of life (65). During this hard time, healthcare workers are the ones that stand on the front line in delivering care to patients. They are usually extremely devoted and dedicated to their work at critical times. However, according to the research, healthcare workers' quality of life is negatively impacted by various factors during pandemics. These include mental and physical health problems such as depression and anxiety, concerns about viral transmission while caring for hospitalized patients and burdens placed on families due to their uncertain health status. These issues, directly and indirectly, are affecting the quality of life for healthcare workers worldwide. Hence, all public and private health institution stakeholders must ensure that healthcare workers have an excellent quality of life.

In our study, multiple linear regression revealed a decline in physical health domain of quality of life between 25 and 35 years of age. We can attribute this to the pre-existing medical condition that begins to manifest in this proportion of responses. In the psychological health domain of quality of life, we observed a positive correlation between quality of life scores and education level among the respondents. These were consistent with the other studies done over the world (66, 67). This is probably because higher education levels can improve financial stability and overall quality of life. Therefore, healthcare workers who received higher education likely experience a better lifestyle due to their higher income, which ensures a higher environmental quality of life. We can prove this as we observed a favorable correlation between the financial solvency of respondents and their environmental domain of quality of life.

This study identified several limitations. Due to the cross-sectional nature of our study, we are unable to determine the temporal relationship between outcome exposure. We did not examine any co-morbid conditions other than psychological distress, religion, or family support that can affect burnout, resilience and quality of life in our study. The study of causality and effectiveness related to improving resilience and quality of life among healthcare workers are recommended for the future studies. Further, the study finding may not have enough confidence on the back of imbalance sample, such as sample had only 51 males as compared to 343 females and similarly, 55 doctors alongside to 248 nurses.

## Conclusion

This study used three separate inventories namely MBI, WHOQOL -BREF questionnaire, and BRS to explore burnout, quality of life and resilience, respectively. Lower physical health was reported among healthcare workers aged 25–35 years, poor psychological health was reported by those who were working for more than 10 h every day and environmental level was affected by income level and working hours of the respondents. Findings from this study call for specific interventions. High correlation between physical and psychological health signifies that if a respondent is facing physical health issues, then it is likely that he/she might face some psychological health issues. Therefore, it is important to improve physical as well as psychological wellbeing of the healthcare workers. Further, negative correlation was found between occupational exhaustion and the four domains of quality of life. Higher score in occupational exhaustion is associated with lower score in quality of life. Therefore, it is important to understand the contribution of long working hours in declining the quality of life. Thus, allocating fixed working hours for healthcare workers would bring a much required change. In the future, we need to take swift action if we're going to respond to COVID-19. It's important to involve a variety of community stakeholders, like traditional and religious leaders, opinion leaders, and healthcare workers. Each country's national government should build its capacity while receiving support from other organizations to ensure the safe integration of COVID-19 outbreak response activities and improve healthcare workers' mental health.

## Data availability statement

The original contributions presented in the study are included in the article/supplementary material, further inquiries can be directed to the corresponding authors.

## Ethics statement

The studies involving human participants were reviewed and approved by Ethics Committee of Management and Science University. The patients/participants provided their written informed consent to participate in this study.

## Author contributions

All authors listed have made a substantial, direct, and intellectual contribution to the work and approved it for publication.

## Funding

This work was supported by the Special Projects of the Central Government Guiding Local Science and Technology Development, China [No.2021L3018]. The funder was not involved in study design, in the collection, analysis and interpretation of data; in the writing of the manuscript; nor in the decision to submit the manuscript for publication.

## Conflict of interest

The authors declare that the research was conducted in the absence of any commercial or financial relationships that could be construed as a potential conflict of interest.

## Publisher's note

All claims expressed in this article are solely those of the authors and do not necessarily represent those of their affiliated organizations, or those of the publisher, the editors and the reviewers. Any product that may be evaluated in this article, or claim that may be made by its manufacturer, is not guaranteed or endorsed by the publisher.
